# NAMPT knockdown attenuates atherosclerosis and promotes reverse cholesterol transport in ApoE KO mice with high-fat-induced insulin resistance

**DOI:** 10.1038/srep26746

**Published:** 2016-05-27

**Authors:** Shengbing Li, Cong Wang, Ke Li, Ling Li, Mingyuan Tian, Jing Xie, Mengliu Yang, Yanjun Jia, Junying He, Lin Gao, Guenther Boden, Hua Liu, Gangyi Yang

**Affiliations:** 1 Department of Endocrinology, the Second Affiliated Hospital, Chongqing Medical University, 400010 Chongqing, China; 2 The Key Laboratory of Laboratory Medical Diagnostics in the Ministry of Education and Department of Clinical Biochemistry, College of Laboratory Medicine, Chongqing Medical University, 400010 Chongqing, China; 3 Department of Endocrinology, the Affiliated Hospital, Zunyi Medical College, 563003 Guizhou, China; 4 The Division of Endocrinology/Diabetes/Metabolism and the Clinical Research Center, Temple University School of Medicine, Philadelphia, Pennsylvania, USA; 5 Department of Pediatrics, University of Mississippi Medical Center, 2500 North State Street, Jackson, Mississippi, MS 39216-4505, USA

## Abstract

NAMPT has been suggested association with atherosclerosis and insulin resistance. However, the impact of NAMPT on atherosclerosis remained unknown. Therefore, the objective of this study was to use a NAMPT loss-of-function approach to investigate the effect of NAMPT on atherosclerosis in hypercholesterolemic mice. We demonstrated that a specific NAMPT knockdown increased plasma HDL-C levels, reduced the plaque area of the total aorta en face and the cross-sectional aortic sinus, decreased macrophage number and apoptosis, and promoted RCT in HFD-fed ApoE KO mice. These changes were accompanied by increased PPARα, LXRα, ABCA1 and ABCG1 expressions in the liver. NAMPT knockdown also facilitated cholesterol efflux in RAW264.7 cells. We further investigated the effect of NAMPT knockdown on the PPARα-LXRα pathway of cholesterol metabolism with MK886 (a selective inhibitor of PPARα) in RAW264.7 macrophages. MK886 abolished the ability of NAMPT knockdown to decrease intracellular cholesterol levels to enhance the rate of ^3^H-cholesterol efflux and to increase ABCA1/G1 and LXRα expressions in RAW264.7 macrophages. Our observations demonstrate that NAMPT knockdown exerted antiatherogenic effects by promoting cholesterol efflux and macrophage RCT through the PPARα- LXRα- ABCA1/G1pathway *in vitro* and *in vivo*.

Nicotinamide phosphoribosyltransferase (NAMPT) was originally identified as pre-B cell colony enhancing factor (PBEF)^1^. Later, NAMPT was thought to be secreted predominantly by visceral adipose tissue and to improve insulin sensitivity *in vivo*^2^,^3^. More recently, NAMPT has been identified as an enzyme involved in NAD biosynthesis^4^ and postulated to play an important role in the regulation of cellular metabolism and insulin secretion^5^,^6^. However, clinical studies have provided controversial results concerning the role of NAMPT in obesity and glucose metabolism^7^,^8^,^9^,^10^,^11^. Therefore, it is currently unclear if NAMPT is involved in the development of insulin resistance (IR) or atherosclerosis.

It has also been reported that NAMPT expression is increased in unstable coronary artery plaques in humans. Immunohistochemical analysis of unstable plaques revealed higher NAMPT concentration compared to stable plaques^2^. It has also been shown that NAMPT induced the release of inflammatory cytokines, cholesterol accumulation and the formation of macrophage foam cells^12^,^13^,^14^. In addition, we and others have reported that NAMPT is involved in the regulation of serum cholesterol metabolism^3^. These studies suggest an association between NAMPT and atherosclerosis. Unfortunately, a homozygous knockout model for the NAMPT gene was incompatible with life, with death occurring in the initial phase of embryogenesis. The lack of knock-out mouse of NAMPT has made it difficult to systematically investigate the role of NAMPT in the development of IR and atherosclerosis.

In the present study, we have taken advantage of the relative tissue specificity of adenovirus for liver and the genetic specificity of shRNA-mediated RNAi to create a relative liver-specific deficiency of NAMPT expression, and examined the role of NAMPT in IR and atherosclerosis by using it to inhibit NAMPT expression in mice.

## Results

### Expressions of NAMPT mRNA and protein in liver and plasma

**Figure 1:**
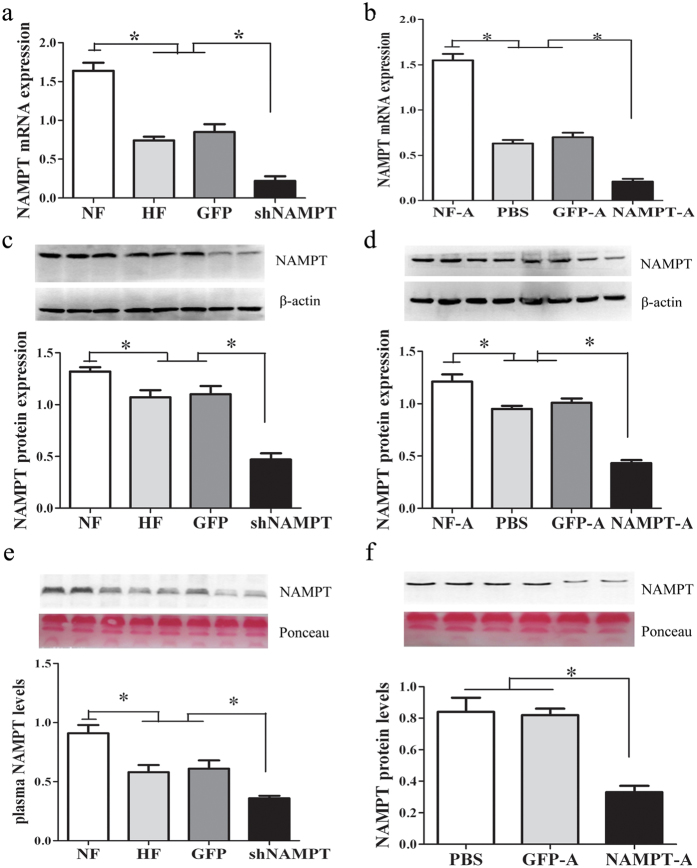
Treatment with Ad-*sh*NAMPT decreases liver expression and plasma levels of NAMPT. (**a**) Hepatic NAMPT mRNA expression in C57BL/6J mice. (**b**) Hepatic NAMPT mRNA expression in ApoE KO mice. (**c**) Hepatic NAMPT protein expression in C57BL/6J mice. (**d**) Hepatic NAMPT protein expression in ApoE KO mice. (**e**) Plasma NAMPT levels in C57BL/6J mice. (**f**) Plasma NAMPT levels in ApoE KO mice. Data are means ± SE. n ≥ 8 per group. *P < 0.01. NF, standard chow diet-fed C57BL/6J mice; HF, high fat diet-fed C57BL/6J mice; GFP, high fat diet -fed C57BL/6J mice treated with Ad-GFP; *sh* NAMPT, high fat diet-fed C57BL/6J mice treated with Ad-*sh* NAMPT; NF-A, standard chow diet-fed ApoE KO mice; PBS, high fat diet-fed ApoE KO mice treated with PBS; GFP-A, high fat diet -fed ApoE KO mice treated with Ad-GFP; NAMPT-A, high fat diet-fed ApoE KO mice treated with Ad-*sh* NAMPT.

A previously validated shRNA sequence against NAMPT was engineered into an adenovirus vector (Ad-*sh*NAMPT) and tested for relative silencing efficiency compared with Ad-GFP. In HFD-fed C57BL/6J mice, the expressions of NAMPT mRNA and protein were reduced by 55% and 25% respectively in the liver compared with normal-chow controls (Fig. 1a,c), whereas Ad- *sh*NAMPT treatment, but not Ad-GFP, achieved a ~71% and ~57% reduction in NAMPT mRNA and protein expressions respectively in the liver (both *P* < 0.01; Fig. 1a,c). When compared with the HF and GFP groups, Ad-*sh*NAMPT treatment also led to a 52% reduction in plasma NAMPT levels (*P* < 0.01, Fig. 1e), demonstrating the efficacy of Ad- *sh*NAMPT *in vivo*. Similarly, in HFD-fed ApoE KO mice (PBS group), the mRNA and protein expressions of NAMPT in the liver were significantly decreased compared with those of SCD-fed ApoE KO mice (NF-A group) (both *P* < 0.01, Fig. 1b,d). Importantly, the mRNA and protein expressions of NAMPT in the liver and NAMPT plasma levels were further decreased by Ad-*sh* NAMPT treatment (all *P* < 0.01, Fig. 1b,d,f).

### NAMPT knockdown fails to affect HFD-induced insulin resistance

We next assessed the effects of NAMPT knockdown on metabolic parameters. Values of body weight and fasting blood glucose (FBG), lipid and fasting insulin (FIns) levels were significantly higher in HFD-fed C57BL/6J mice. HDL-C and FIns in Ad-*sh* NAMPT treated plus HFD-fed mice were significantly higher than those of their littermates fed HFD alone (Supplemental Table S1). To assess the effects of NAMPT knockdown on insulin sensitivity, euglycemic- hyperinsulinemic clamps (EHCs) were performed. As shown in Supplemental Table S2, during the clamps, TG, TC, FFA and HDL-C levels were suppressed by hyperinsulinemia in four groups, but HDL-C remained higher in the Ad-*sh* NAMPT group than in the other three groups (*P* < 0.01). The glucose infusion rate (GIR) required was 50% lower in HFD-fed mice compared with SCD-fed controls. Ad-*sh* NAMPT treatment had no effect on the GIR and glucose disposal rate (GRd). In addition, the ability of insulin to suppress hepatic glucose production (HGP) was unchanged in the HFD-fed plus Ad-*sh*NAMPT treated mice compared with HFD-fed mice alone (Supplemental Table S2).

### Effect of NAMPT knockdown on metabolic parameters in HFD-fed ApoE KO mice

As shown in Table 1 and Supplemental Fig. S1, Ad-*sh*NAMPT treatment in HFD-fed ApoE KO mice significantly increased HDL-C and apoA-I levels compared with PBS and Ad-GFP treatment. However, there were no differences in the other parameters. In addition, Ad-*sh*NAMPT treatment decreased hepatic TC content to 76% of that in ApoE KO mice fed HFD (*P* < 0.05), whereas hepatic TG content remained unchanged (Table 1).

### NAMPT knockdown markedly inhibits the formation of atherosclerotic lesions

**Figure 2:**
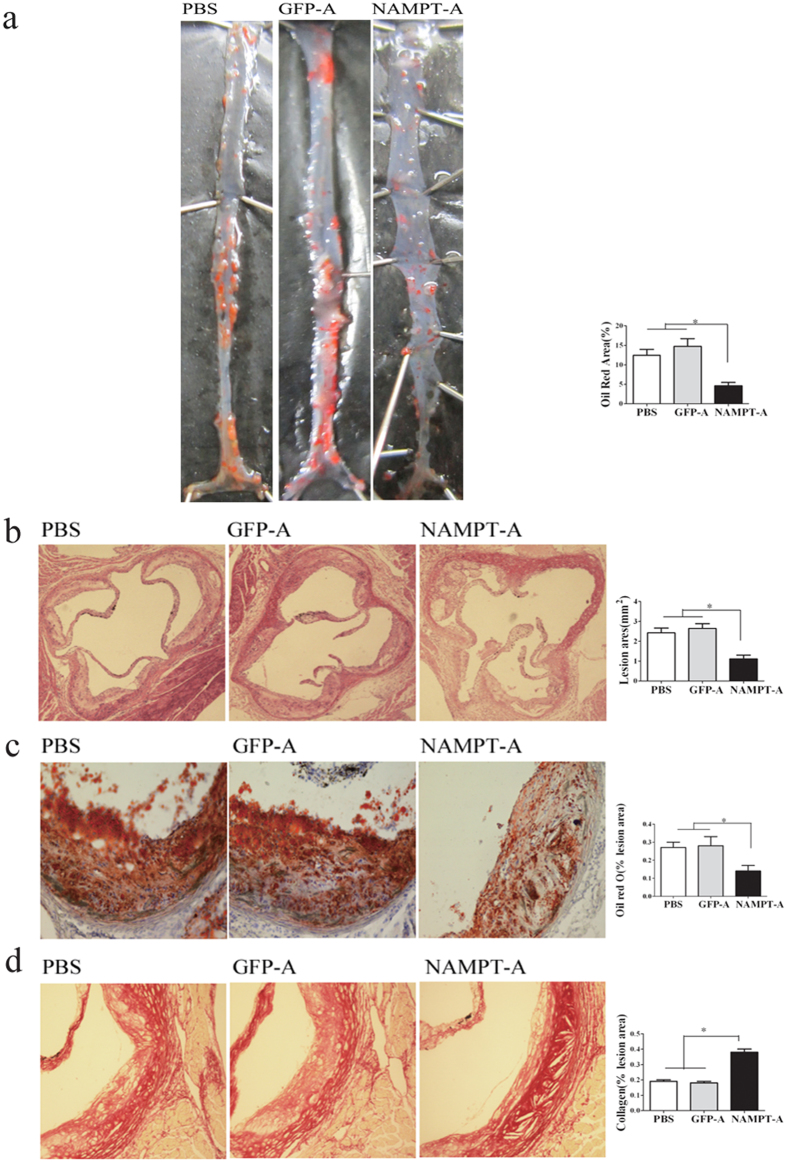
Ad-*sh*NAMPT administration attenuates atherosclerotic lesion in the entire aorta and aortic roots of ApoE KO mice. (**a**) Representative Oil Red O stained *en face* aortae from ApoE KO mice (left) and quantitative assessment of plaque surface area in the entire aorta (right). (**b**) Representative H&E stained aortic root cross sections from ApoE KO mice (left) and quantitative assessment of plaque area in the aortic root (right). (**c**) Representative Oil Red O stained aortic root cross sections for the assessing of lipid deposition in ApoE KO mice (left) and quantitative assessment of the plaque area (right). (**d**) Representative picrosirius red stained aortic root cross sections for the assessing of collagen contents in ApoE KO mice (left) and quantitative assessment of the plaque area (right). Data are the mean ± SE. n = 10 per group. Original magnification: ×40(B), *P < 0.01.

**Figure 3:**
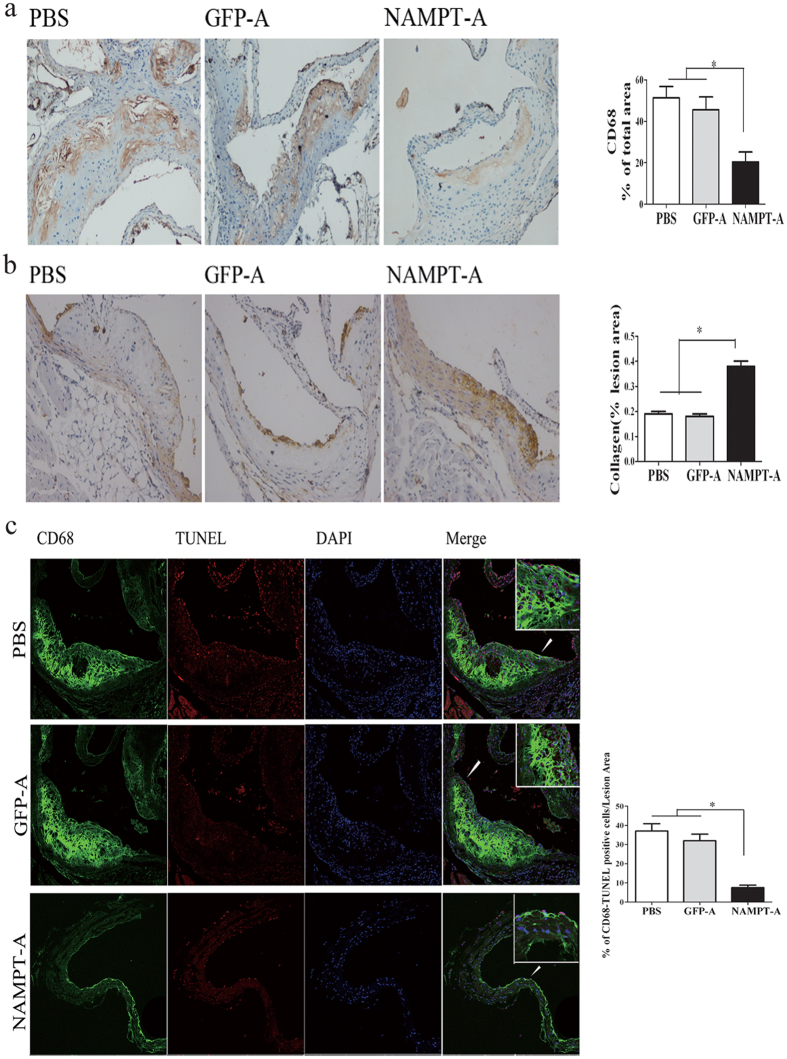
Ad-*sh*NAMPT administration improves a more stable phenotype of atherosclerotic lesions. (**a**) Representative photomicrographs of aortic roots from ApoE KO mice stained with an antibody against CD68 for macrophages (left) and quantitative assessment of the plaque area (right). (**b**) Representative immunohistochemical staining of aortic roots from ApoE KO mice for α-SMA (left) and quantitative assessment of the plaque area (right). (**c**) Apoptotic cells in the lesion area were detected by TUNEL staining, and macrophages by CD68 staining. Representative images (×20 magnification) are shown left and quantitative data are shown right. Data are the mean ± S.E. Magnification ×200, *P < 0.01.

As shown in Supplemental Fig. S2, immunohistochemistry found that the expression of NAMPT in the atherosclerotic plaques of Ad-*sh*NAMPT mice was significantly decreased compared with that of Ad-GFP or PBS mice (*P* < 0.01), suggesting that NAMPT signaling was inhibited. To investigate the effects of NAMPT knockdown on the formation of atherosclerotic plaques, atherosclerotic lesions in the aorta were analyzed by en face analysis of the spread total aorta and analysis of cross sections of the aortic sinus. The en face plaque area of the total aorta stained with oil red O was reduced by 67% in HFD-fed and Ad- shNAMPT treated ApoE KO mice compared with HFD-fed ApoE KO mice alone (Fig. 2a). In addition, the cross- sectional plaque area of the aortic sinus stained with H&E was reduced by 54% in Ad- HFD-fed and *sh*NAMPT-treated ApoE KO mice compared with HFD-fed mice alone (Fig. 2b). In Ad-*sh*NAMPT treated mice, oil red O staining also revealed a ~50% decrease in lipid deposition of aortic sinus lesions, whereas picrosirius red staining showed a 2-fold increase in collagen content in plaque areas (Fig. 2c,d). To investigate whether NAMPT knockdown also affects the plaque phenotype, we examined two key parameters as surrogate markers for lesion vulnerability. We found that plaques from aortic root of Ad- *sh*NAMPT-treated ApoE KO mice exhibited fewer CD68^+^ macrophages as compared with control littermates (Fig. 3a). In agreement with that, the presence of α-SMA positive cells was increased in Ad- *sh*NAMPT-treated mice (Fig. 3b) as was the accumulation of collagen. Meanwhile, immunofluorescent costaining with CD68 and TUNEL displayed a decrease in the percentage of CD68/TUNEL positive cells in the atherosclertic plaque of Ad-*sh*NAMPT mice (Fig. 3c).

### Effect of NAMPT knockdown on macrophage cholesterol efflux

**Figure 4:**
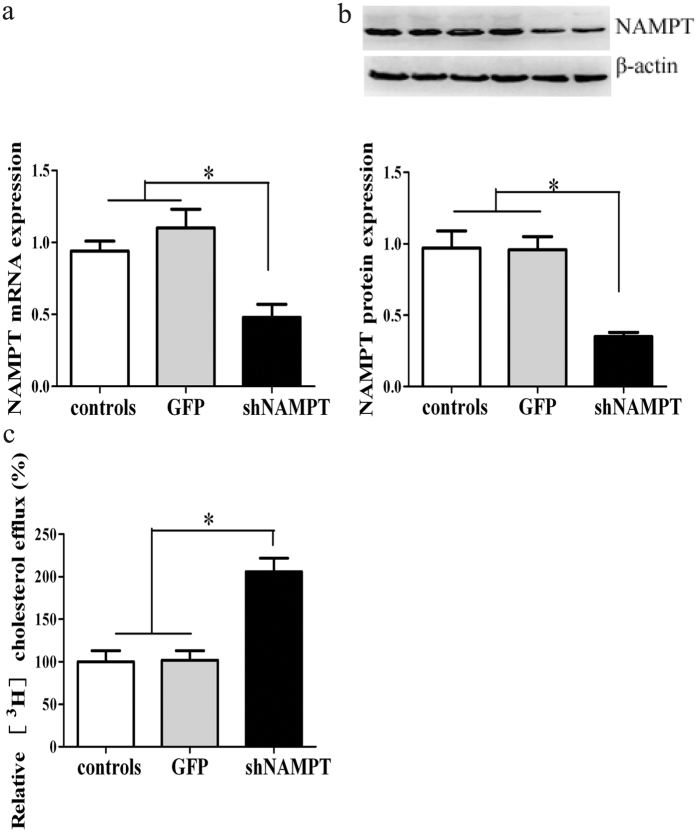
NAMPT knockdown promotes the HDL-mediated cholesterol efflux in RAW cells. (**a**) NAMPT mRNA expression in RAW cells. (**b**) NAMPT protein expression in RAW cells. (**c**) The HDL-mediated cholesterol efflux in RAW cells. The efflux of untreated cells represents is expressed as 100%. The data represent mean ± SE in three independent experiments. ^*^P < 0.01. Controls, RAW264.7 cells; GFP, RAW cells treated with Ad-GFP; *sh*NAMPT, RAW cells treated with Ad-*sh*NAMPT.

We loaded macrophages with ^3^H cholesterol plus HDL and determined HDL- mediated efflux of cholesterol by measuring the appearance of cholesterol in the medium. NAMPT treatment decreased ^3^H cholesterol release in a concentration-dependent fashion (Supplemental Fig. S3). To determine if NAMPT deficiency influences macrophage cholesterol efflux, we conducted *in vitro* assays testing effects of Ad-*sh*NAMPT treatment on cholesterol efflux from ^3^H-cholesterol-loaded RAW cells to lipoprotein acceptors. In Ad-*sh*NAMPT -treated cells, both NAMPT mRNA and protein expressions were significantly decreased (Fig. 4a,b). Ad-*sh*NAMPT treatment promoted the HDL-mediated efflux of ^3^H- cholesterol from labeled RAW cells by 106%, suggesting that NAMPT deficiency is able to activate the cholesterol efflux that exports excess cholesterol from macrophages (Fig. 4c).

### NAMPT knockdown increases HDL-C and enhances RCT *in vivo*

**Figure 5:**
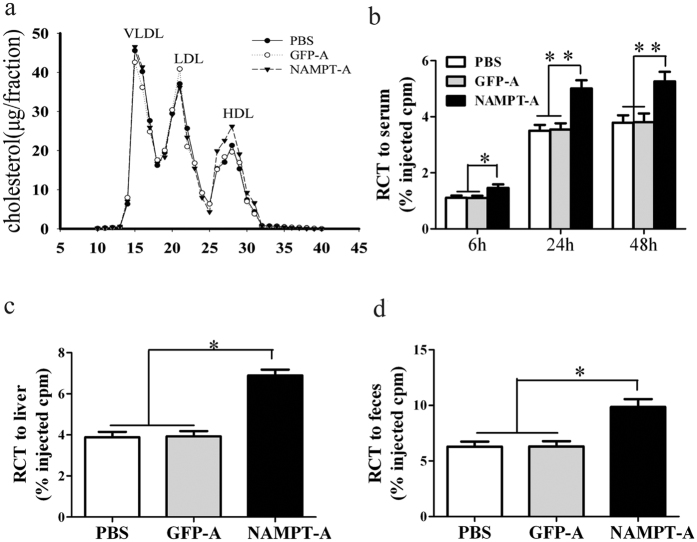
NAMPT deficiency increases plasma HDL-C concentration and promotes RCT in HFD-fed ApoE KO mice. (**a**) FPLC analysis of plasma lipoprotein distribution from ApoE KO mice. Each profile represents a separation of a plasma pool from 8 mice of each group. (**b**) Time course of ^3^H-cholesterol distribution in plasma. (**c**) Hepatic ^3^H-cholesterol tracer levels after 48 hours. (**d**) Fecal ^3^H-cholesterol tracer levels. Feces were collected continuously from 0 to 48 hours after injection. Data are expressed as the percentage of the ^3^H-cholesterol tracer relative to total cpm tracer injected ± SE. *P < 0.05, **P < 0.01. RCT, macrophage reverse cholesterol transport; HLD-C, High-density lipoprotein cholesterol; other abbreviations as in Fig. 1.

We next analyzed the lipoprotein profiles in plasma from three groups of mice by FPLC. The results showed that the increased plasma cholesterol in Ad-*sh*NAMPT mice was mainly in the HDL fractions (column fractions 26–31), with no difference in the VLDL and LDL regions (Fig. 5a).

To determine whether Ad-*sh*NAMPT treatment triggers higher HDL-C levels in response to upregulation of cholesterol transport from peripheral cells to the liver for further excretion into bile, we performed an *in vivo* RCT assay that traces ^3^H-cholesterol derived from macrophages loaded with cholesterol *ex vivo*. After intraperitoneal injection of ^3^H-cholesterol- labeled macrophages, plasma ^3^H-cholesterol levels at 6, 24 and 48 h after injection were significantly higher in Ad-*sh*NAMPT mice compared with PBS- and Ad-GFP-treated mice (Fig. 5b). Ad-*sh*NAMPT mice showed a 44% increase in the delivery of macrophage-derived ^3^H-tracer to the liver and a 35% increase in ^3^H-sterols excreted into feces (Fig. 5c,d). Therefore, NAMPT inhibition not only increases circulating HDL-C, but also enhances the RCT pathway by which excess cholesterol is effluxed from peripheral tissues.

### Effects of NAMPT knockdown on gene expressions related to cholesterol metabolism in the liver

**Figure 6:**
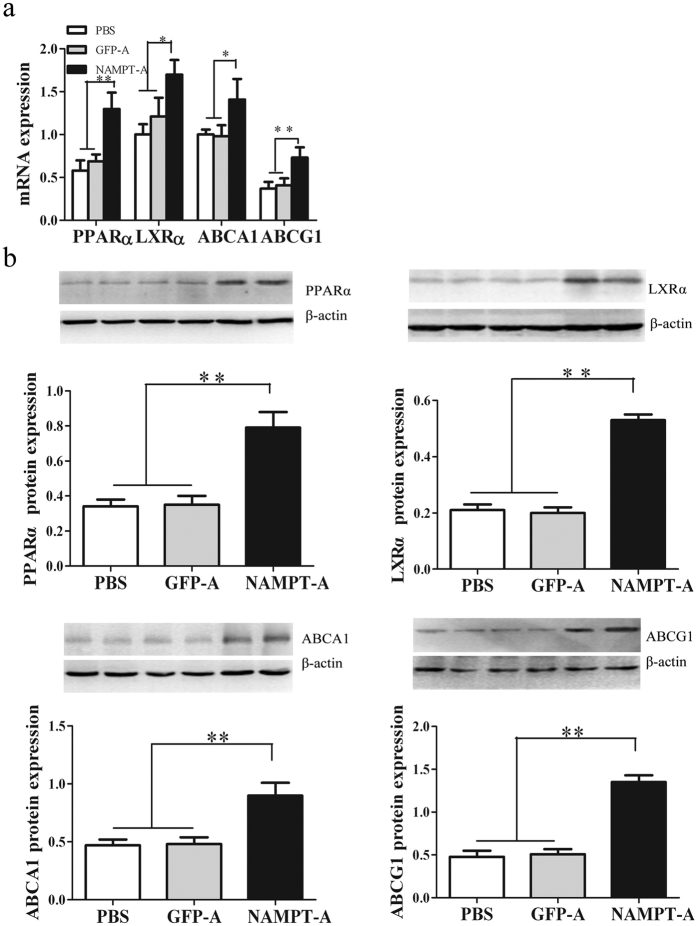
The effects of NAMPT deficiency on mRNA and protein expression of hepatic genes involved in cholesterol metabolism in HFD-fed ApoE KO mice. (**a**) Analysis of hepatic gene expression by real-time quantitative PCR. (**b**) Analysis of hepatic gene expression by Western blots. Data are means ± SE. n = 5 per group. *P < 0.05, **P < 0.01. PPARα, Peroxisome proliferator-activated receptors-α; LXRα, Liver X receptor-α; ABCA1, ATP binding cassette transporters A-1; ABCG1, ATP binding cassette transporters G-1.

Next, we found that the PPARα, LXRα, ABCA1 and ABCG1 mRNA and protein contents in the liver were markedly increased in Ad-*sh*NAMPT treated mice compared with control mice (Fig. 6a,b).

### NAMPT knockdown-induced reduction in cholesterol levels and the expression of PPARα is blocked *in vitro* by MK886

**Figure 7:**
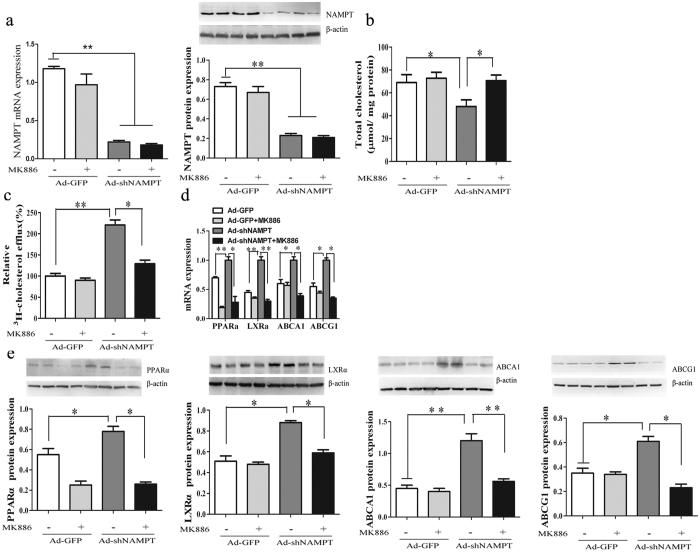
NAMPT deficiency stimulates the expression of LXRα, ABCA1 and ABCG1 and decreased cholesterol levels via PPARα-dependent mechanisms. RAW cells were transduced with Ad-GFP or Ad-*sh*NAMPT in the presence or absence of MK886. (**a**) NAMPT mRNA (lift) and protein expression (right). (**b**) Cellular cholesterol levels. (**c**) The HDL-mediated cholesterol efflux. (**d**) PPARα, LXRα, ABCA1 and ABCG1 mRNA expression. (**e**) PPARα, LXRα, ABCA1 and ABCG1 protein expression. Data represent the mean ± SE from three independent experiments. *P < 0.05, **P < 0.01. MK886, a selective inhibitor of PPARα.

Because of the well-documented stimulatory role of PPAR-α activators on hepatic HDL production and ABCA1-mediated cholesterol efflux from macrophages^15^, we investigated the effects of Ad-*sh*NAMPT on cholesterol metabolism and the PPARα/LXRα/ABCA1/ABCG1 pathway in RAW cells. Ad-*sh*NAMPT treatment in RAW cells led to an 81% and a 68% decrease respectively in the expression of NAMPT mRNA and protein (Fig. 7a). We further studied the effects of NAMPT knockdown on cholesterol metabolism and the expression of genes involved in cholesterol efflux and the RCT pathway in these cells. In RAW cells, Ad-*sh*NAMPT led to a 33% decrease in total cholesterol levels (Fig. 7b). However, this effect was negated by the coadministration of MK886, a selective inhibitor of PPARα (Fig. 7b). Ad- *sh*NAMPT treated cells resulted in a 121% increase of ^3^H-cholesterol efflux to HDL compared to that of untreated cells, whereas, this effect was inhibited by MK886 (Fig. 7c). Consistent with the changes observed for cholesterol levels, ABCA1, ABCG1, PPARα and LXRα mRNA and protein were significantly increased in Ad-*sh*NAMPT treated cells (Fig. 7d,e). Importantly, these changes were negated by MK886 treatment (Fig. 7d,e). These findings suggest that the effect of NAMPT on cholesterol metabolism may be mediated via the PPARα/LXRα/ABCA1/ABCG1 signaling.

## Discussion

The absences of NAMPT knock-out models have severely hindered the study of NAMPT’s function. To better understand the role of NAMPT deficiency in IR and atherosclerosis, we have developed an adenovirus- mediated RNA interference technique to knock down NAMPT expression in *vivo*. In the present study, we have taken advantage of the relative tissue specificity of adenovirus for liver to create a relative liver-specific deficiency of NAMPT^16^. This technique allowed us to investigate the role of NAMPT deficiency without the lifelong loss of function^16^. Although systemic administration of adenoviral vectors can cause the inflammatory immune response and cytotoxicity, numerous studies have demonstrated that third-generation adenoviral vectors, as used in this study, are superior to first-and second- generation adenoviral vectors in terms of mediating long-term transgene expression with negligible inflammatory immune response and hepatotoxicity^17^,^18^. In support of this notion, we previously reported that our adenoviral vectors failed to increase inflammatory cytokine expressions and inflammation revealed by hematoxylin and eosin histology in the liver^19^.

The adenovirus-mediated RNAi resulted in high silencing efficiency and long lasting effects (up to 4 weeks with 2 injections)^20^. This allowed us to evaluate the metabolic and arteriosclerotic impact of hepatic NAMPT knockdown in an IR animal model. Unexpectedly, HFD-fed mice with reduced hepatic NAMPT content showed no alterations in any of the tested metabolic parameters, except for elevated HDL-C and slightly decreased LDL-C levels which were not statistically significant. We then performed EHCs for an in-depth analysis of the effects of NAMPT knockdown on insulin sensitivity. Surprisingly, these clamp studies showed that GIR, HGP, and GRd were unchanged in HFD-fed mice treated with Ad-*sh*NAMPT suggesting that NAMPT knockdown did not impact insulin sensitivity *in vivo*.

However, we noted a trend of elevated HDL-C and apoA-I levels in HFD-fed mice with NAMPT knockdown. This led us to investigate whether NAMPT knockdown affected cholesterol metabolism and the development of atherosclerosis. For this purpose, we treated HFD-fed ApoE KO mice with Ad-*sh*NAMPT and examined metabolic parameters and atherosclerotic lesions. We observed that plasma HDL-C levels in HFD-fed ApoE KO mice treated with Ad-*sh*NAMPT were significantly increased and hepatic TC contents were decreased compared to littermates fed HFD alone. Analysis of lipoproteins showed an increase in cholesterol content in the HDL fractions of Ad- *sh*NAMPT-treated mice, suggesting that NAMPT may be important for cholesterol metabolism. Currently, it is widely accepted that even subtle changes in HDL cholesterol may have profound effects on the development of atherosclerosis. Therefore, we investigated the effects of *in vivo* NAMPT on atherosclerotic lesions in the aorta. We found that NAMPT deficiency attenuated atherosclerotic lesions in the entire aorta and the aortic sinus and reduced lipid accumulation in aortic sinus lesions. Although lesion size accurately reflects atherosclerosis progression, plaque phenotype is a more important predictor of plaque disruption and acute clinical events in humans^21^. Therefore, we evaluated several key parameters as surrogate markers for lesion vulnerability. Our findings indicate that NAMPT knockdown in ApoE KO mice decreased lipid content, CD68^+^ macrophages, macrophage apoptosis, and increased α-SMA positive cells and the accumulation of collagen in atherosclerotic plaque. These data indicate that NAMPT knockdown reduced atherosclerotic plaque macrophage infiltration and lesion burden by modifying plaque composition, which reflected a stable atherosclerotic plaque phenotype. Here, NAMPT knockdown seemed to exert contradictory effects in these experiments, protecting against atherosclerosis without changing insulin sensitivity. Therefore, we speculated that the reduction of hepatic NAMPT expression in both HFD-fed C57BL/6J and ApoE KO mice might be a compensatory down-regulation to counteract the metabolic stress imposed by obesity or abnormal cholesterol metabolism. This down-regulation may play a role in preventing atherosclerotic progression. We also considered the possibility that the antiatherogenic effects of NAMPT knockdown could have been due to other factors, such as macrophage recruitment, cholesterol efflux to HDL and changes in RCT. Consequently, we investigated the effect of NAMPT knockdown on these factors.

The RAW 264.7 cell line is a model widely used to study macrophage regulation of cholesterol efflux. We found that in cultured RAW264.7 cells, HDL-mediated ^3^H-cholesterol efflux was increased by NAMPT knockdown, suggesting that NAMPT knockdown led to an increase in the ability of cells to efflux intracellular cholesterol to HDL and therefore to promote the initial step of macrophage RCT. However, this observation contradicted previous findings by Dahl T *et al*.^22^, who reported that a reduction in cholesterol efflux in ox-LDL-exposed macrophages was induced by FK866, a pharmacological inhibitor which mediated inhibition of intracellular NAMPT. The reason for this difference is not clear but may be related to: 1) differences in study protocol, conditions and methods. For example, their studies were performed with ^3^H-cholesterol and ox-LDL treated THP-1 cells, whereas we used ^3^H-cholesterol-treated RAW264.7 cells; 2) the different pharmacological and molecular inhibition of NAMPT; 3) the inability of FK866 to block NAMPT from triggering the IL-6/STAT3 cell survival pathway^23^ and lastly, 4) ox-LDL has been reported to enhance NAMPT expression in THP-1 cells^22^. Thus, further studies are needed to confirm the role of NAMPT in cholesterol efflux.

As RCT is believed to be a critical mechanism by which HDL-C exerts its protective effects on atherosclerosis, we performed an *in vivo* RCT assay that traces ^3^H-cholesterol from macrophages, loaded *ex vivo* with cholesterol. Consistent with the *in vitro* cholesterol efflux, results of the RCT showed that higher HDL-C levels in response to NAMPT knockdown increased cholesterol transport from peripheral macrophages to the liver. Therefore, NAMPT inhibition not only increased circulating HDL-C, but also enhanced RCT by which excess cholesterol is effluxed from peripheral tissues, a process that is particularly important in the removal of cholesterol from atherosclerotic lesions. These results also suggest that reduced circulating NAMPT produced from the liver and/or other tissue might be the main explanation for increased cholesterol efflux from the injected RAW cells. As the outcome of numerous studies has shown that the rate of macrophage RCT has a greater influence on the degree of atherosclerosis than the levels of HDL-C^24^, this increase in RCT could be primarily responsible for the positive effects of NAMPT knockdown on atherosclerosis. These data establish NAMPT inhibition as an attractive therapeutic target for raising HDL-C, increasing RCT, and reducing atherosclerosis.

It has been shown that PPARα activation enhances cholesterol efflux and RCT in association with increased expression of ABCA1/ABCG1 in macrophages via the PPARα-LXR pathway^25^. Understanding how NAMPT is integrated into this complex genetic network may reveal novel therapeutic targets for antiatherosclerotic treatment. To better understand the impact of NAMPT on gene expression involved in cholesterol efflux and RCT, we measured the expression of ABCA1, ABCG1, PPARα and LXRα in the liver. The results demonstrated that NAMPT knockdown upregulated mRNA and protein expressions of these genes. Consistent with these observations, Rotllan *et al*.^26^ reported that the PPARα agonist fenofibrate promoted macrophage RCT and enhanced cholesterol efflux and ABCA1/G1 expression in primary macrophages. Together, these observations suggest that NAMPT inactivation promotes cholesterol efflux and macrophage RCT via a complex pathway involving ABCA1/G1, PPARα and LXRα. These results support the concept that promotion of macrophage RCT may be one mechanism by which NAMPT knockdown inhibits the development of atherosclerosis in hypercholesterolemic mice. Moreover, our present data suggested that an important factor in the promotion of overall RCT and cholesterol efflux by NAMPT knockdown was activation of PPARα. We, therefore, examined whether PPARα plays a role in the promotion of RCT by NAMPT knockdown by using MK886 (a selective inhibitor of PPARα) in RAW cells. Noteworthy, MK-886 is also an inhibitor of leukotriene biosynthesis. Prolonged treatment with the 5-lipoxygenase inhibitor MK-886 reduced development of atherosclerosis by inhibiting inflammatory responses in animal model^27^,^28^. Therefore, the role of MK886 may be a bilateral regulator for inflammatory responses and atherosclerosis^29^,^30^. In addition, MK886 has been shown to be a non-competitive inhibitor of PPARα activity. It inhibits PPARα activity though its impact on the transcriptional levels of PPARα promote-driven reporter^31^,^32^,^33^,^34^. In the current study, we found that MK886 negated NAMPT knockdown-induced ABCA1/G1 and LXRα expressions in these cells. Moreover, MK886 treatment negated the effect of NAMPT knockdown on intracellular cholesterol levels and the rate of ^3^H-cholesterol efflux. Collectively, our *in vivo* and *ex vivo* findings are consistent with the concept that NAMPT knockdown enhances cholesterol efflux in association with increased expression of ABCA1/G1 in the liver, resulting in promotion of overall RCT via the PPARα-LXRα pathway.

During the preparation of our paper, Nencioni *et al*. reported that treatment with FK866, a NAMPT inhibitor, reduced neutrophil infiltration and MMP-9 content and increased collagen levels in atherosclerotic plaques in ApoE KO mice^35^. However, they did not examine effects of NAMPT knockdown on insulin sensitivity, RCT, HDL-mediated ^3^H-cholesterol efflux or lipoprotein profiles. They also did not evaluate the en face plaque area of the total aorta. Importantly, the mechanism linking NAMPT knockdown to cholesterol metabolism was not delineated in that study.

In summary, this study has provided new information with respect to the role of NAMPT in the development of atherosclerosis. Our findings are schematically presented in Supplemental Fig. S4. Our results indicate that NAMPT knockdown exerts antiatherogenic effects by promoting macrophage RCT through the PPARα-LXRα pathway, and is consistent with the concept that NAMPT may be a negative regulator of macrophage RCT and the development of atherosclerosis in humans. Therefore, these findings not only reveal a novel function of NAMPT in regulating lipid metabolism and preventing plaque formation, but also underscore as an important therapeutic target for atherosclerosis and cardiovascular diseases.

## Methods

### Ethics Statement

Animal experiments were performed in adherence with the US National Institutes of Health Guidelines on the use of laboratory animals (NIH Publication No. 85–23, revised 1996) and were approved by the Chongqing Medical University Committee on Animal Care.

### Generation of NAMPT knockdown vector

To construct vectors expressing shRNAs against NAMPT, we designed several oligonucleotides and complementary strands to target specifically mouse NAMPT. The sequence 5′-GCGCTTTGCTACAGAAGTTAA -3′ was the most effective one and therefore, was selected for subsequent experiments. A recombinant vector Ad-*sh*NAMPT was generated by transfection in 293 cells with the Ad-Easy system. A control recombinant vector Ad-GFP encoding enhanced green fluorescence protein (GFP; Clonetech) was used as a control. Large-scale amplification and purification of recombinant adenoviruses were performed using the ViraBind Adenovirus Purification Kit (Cell Biolabs Inc., San Diego, CA, USA).

### Animal preparation for the *in vivo* experiments

Male C57BL/6J and ApoE KO mice were purchased from The Experimental Animal Center of Beijing University of Medical Sciences (Beijing, China) at 3 weeks of age, and fed either a standard chow diet (SCD, 18% kcal from fat) or a high fat western diet consisting of 42% fat and 0.15% cholesterol (HFD, Research Diets Inc.) for 12 weeks. C57BL/6J mice were injected with Ad- *sh*NAMPT, Ad-GFP (1 × 10^9^ pfu in 100 μl of PBS), or sterile saline by tail vein at week 11 of receiving HFD feeding. At week 8 and 10 of HFD feeding, ApoE KO mice were also treated with Ad- *sh*NAMPT, Ad-GFP or sterile saline by tail vein.

### Hyperinsulinemic-euglycemic clamps

Thirty eight C57BL/6J mice were randomized into four groups: SCD (NF group, n = 10), HFD (HF group, n = 10), HFD plus Ad-GFP (GFP group, n = 8), HFD plus Ad-*sh*NAMPT (*sh*NAMPT group, n = 10). Four days before the clamp studies, animals were anesthetized with ip ketamine (80 mg/kg) and xylazine (16 mg/kg) and catheters were placed into the right internal jugular vein and left carotid artery^36^,^37^. Clamp studies were performed in conscious mice as previously described^37^. In brief, food was removed 8 hours prior to beginning of studies. At *t* = −90 min, a primed, continuous infusion of HPLC-purified [3-^3^H]-glucose (Amersham, Los Angeles, CA, USA; 5 μCi bolus, 0.05 μCi/min) was initiated and continued for 3.5 hours. At 0 min, a continuous infusion of insulin and a variable infusion of 20% glucose were started. Blood samples (50 μl) were obtained from the jugular vein catheter at 0, 80, 90, 100 and 120 min for determination of insulin, free fatty acids (FFA) and glucose specific activity. At the end of the clamp, the mice were anesthetized, and tissue samples were freeze-clamped *in situ* with aluminum tongs, pre-cooled in liquid nitrogen and stored at −80 °C for subsequent analysis.

### Histological analysis

40 ApoE KO mice were divided into three groups: HFD plus saline mice as diet controls (PBS group, n = 10); HFD plus Ad- *sh*NAMPT mice (NAMPT-A group, n = 10) and HFD plus Ad- GFP mice (GFP-A group, n = 10). Mice were killed with an overdose of isoflurane and exsanguinated by cardiac puncture at the end of two weeks after the second vector injection. Mice were then perfused with 4% paraformaldehyde in PBS. Hearts and descending aortas were excised. Hearts were fixed in paraformaldehyde and then either embedded in paraffin or Optimal Cutting Temperature (OCT). The cross-sections (5 μm thick) of aortic sinus embedded in paraffin were stained with Hematoxylin and Eosin (H&E) for histological analysis or with Picrosirius Red for collagen analysis as previously described^38^. For assessing of neutral lipid, the frozen cross-sections (10 μm thick) of aortic sinus embedded in OCT were stained with Oil Red O, and counterstained with hematoxylin. For analyses of aortic lesions en face, Oil Red O staining of the excised aortas from the arch to the common iliac levels was performed after fixation in phosphate-buffered 10% formaldehyde. Percentages of en face Oil Red O positive areas to total aortic areas were calculated. Quantitative analyses were performed by using the Image Pro-Plus software (Media Cybernetics).

### Immunohistochemistry and Immunofluorescence

Paraffin-embedded cross sections of aortic sinus were deparaffinized by immersion in xylene, and were blocked with 5% normal goat serum for 60 min. Sections were incubated with rat anti-mouse NAMPT (1:500; Abcam, Cambridge, UK), CD68 (1:100; Abcam, Cambridge, UK) and alpha smooth muscle actin (α-SMA) (1:500; Abcam, Cambridge, UK) overnight at 4 °C. Sections were then washed and incubated with biotinylated secondary antibody (Co Win Biotech, Beijing, China) for 60 min at room temperature. Antigen-antibody complexes were detected with the Avidin-biotn peroxidase for 60 min at room temperature. Bound antibody was visualized with DAB (Co Win Biotech, Beijing, China). Nuclei were counterstained with hematoxylin. Negative control was carried out by substituting the primary antibody with PBS^39^. For immunofluorescence analyses, the tissue sections were first stained with CD68, followed by TUNEL staining with a situ cell death detection kit (Roche Molecular Biochemicals, Germany) according to the manufacturer’s protocol. The nuclei were stained with 40, 6- diamidino-2-phenylindole (DAPI). Images were obtained using a fluorescence microscope (Nikon Eclipse Ti) or digitized microscope (Olympus) and were analyzed by ImagePro plus software (Media Cybernetics)^40^,^41^. The quantitation of tissue sections was performed by observers blinded to specimen.

### Cell Culture

RAW 264.7 (RAW) macrophages were obtained from the American Type Culture Collection (ATCC, Manassas, VA, USA) and routinely propagated at 37 °C in a humidified atmosphere of 5% CO_2_ in Dulbecco’s modified Eagle’s medium (Gibco, Carsbad, CA) supplemented with 10% fetal bovine serum, 100 μg/ml penicillin, and 100 μg/ml streptomycin.

### Cholesterol Efflux Assay

RAW cells were grown to 60% confluence in 24-well plates, and then incubated with DMEM containing 1 μCi of [1, 2-^3^H] cholesterol per ml for 48 h. RAW cells were washed three times with PBS, and Cellular cholesterol pools were allowed to equilibrate for another 24 h in DMEM containing 0.1% BSA (DMEM-BSA), Ad-GFP or Ad-*sh*NAMPT (10 μl 1 × 10^11^ pfu/ml). For dose-dependent experiments, RAW cells were stimulated with recombinant mouse NAMPT (Alexis Biochemicals, Paris, France) in concentrations of (40, 100 and 200 nM) for another 24 h. After intensive washing of cells with PBS-BSA, Efflux studies (24 h) were carried out using 100 μg of HDL per ml prepared in DMEM-BSA as the cholesterol acceptor. After the efflux period, Medium was collected and centrifuged, and radioactivity was counted by liquid scintillation counting (LSC). The residual radioactivity in the cell fraction was determined after an overnight extraction with hexane-isopropanol (3:2). Percentages of cholesterol efflux to HDL were calculated by dividing the radioactive counts in the efflux medium by the sum of the radioactive counts in the medium and the cell fraction. The values were normalized with untreated control cells expressed as 100%.

### Macrophage *in vivo* reverse cholesterol transport (RCT) study

RCT studies were performed as previously described^42^,^43^. RAW cells were plated in 6-well plates (2 × 10^5^ cells/well) and 24 hours later were labeled with 5 μCi of [1, 2-^3^H] cholesterol and 50 μg/ml acetylated LDL (AcLDL) in DMEM plus 10% FBS media for 48 h. These foam cells were washed twice, equilibrated in DMEM plus 0.2% BSA for 6 h, spun down, and resuspended in DMEM medium before use. The cholesterol content of cells was markedly elevated, and the majority of cellular cholesterol was esterified, as determined by thin-layer chromatography.

^3^H-cholesterol-labeled and AcLDL-labeled RAW cells (typically 4.5 × 10^6^ cells containing 6.4 × 10^6^ counts per minute (cpm) in 0.5 ml DMEM) were injected intraperitoneally into HFD-fed ApoE KO Mice treated with PBS, Ad-GFP or Ad-*sh* NAMPT. Prior to injection, an aliquot of cells was counted using LSC to measure baseline radioactivity. Blood was collected at 6, 24, and 48 h, and plasma was used for LSC. Feces were collected continuously from 0–48 h, homogenized in 50% NaOH overnight and counted in an LSC. At 48 hours after injection, mice were killed with an overdose of isoflurane and exsanguinated by cardiac puncture. Mice were perfused with cold PBS, and portions of the liver were removed. Lipids extractions were performed as described^44^, and radioactivity was counted by LSC. RCT to plasma, feces, and liver was calculated as a percentage of total radioactivities injected at baseline.

### Studies on the Participation of Peroxisome proliferator-activated receptors-α (PPARα) in RAW264.7 cells

RAW cells were grown to 60% confluence in 6-well plates, and then incubated with DMEM containing 1 μCi of [1, 2-^3^H] cholesterol per ml for 48 h. Percentages of cholesterol efflux to HDL were determined as mentioned above in RAW cells treatment with Ad-*sh*NAMPT or Ad-GFP in the presence or absence of the selective inhibitor of PPARα (10 μM, MK886, Cayman Chemical Company). Total cellular cholesterol was measured using cholesterol assay kits purchased from Applygen Technologies Inc (Beijing, China). Expression of NAMPT, PPARα, LXRα, ABCA1 and ABCG1 were determined by Real-time RT-PCR and western blot analysis.

### Real-time RT-PCR analysis

Total RNA was extracted from tissue (100 mg) and cells using Trizol reagent (Invitrogen, Carlsbad, CA, USA). Real time polymerase chain reaction (qRT-PCR) was performed with a SYBR Green PCR kit (Takara Bio Inc. Otsu, Japan), and a Corbett Rotor Gene 6000 real-time PCR system (Corbett Research, Sydney, Australia) according to the manufacturer’s instructions. Gene expressions were analyzed by the comparative Ct method and normalized to β-actin. Forward and reverse primers are listed in Supplemental Table S3.

### Western blot analyses

Tissues were homogenized. For the *in vitro* experiments, cell lysates and medium were collected 48 h after transfection. Protein concentration was measured with a BCA protein quantification kit (Pierce Biotechnology). Plasma samples (50 μl) were diluted 1:10 in PBS, reduced by adding Nupage sample reducing agent (Invitrogen), and heat denatured. Three microliters of the 1:10 diluted samples were loaded on a 10% Novex Bis-Tris gel (Invitrogen). Total protein in plasma was assessed by staining the blot with Ponceau S. Cell lysates or tissue extracts (70 μg) were separated by SDS-polyacrylamide gel electrophoresis and transferred to PVDF membranes. Immunoblots were then blocked in TBS containing 0.1% Tween-20 and 5% skimmed milk overnight at 4 °C, and then incubated with primary antibodies including anti-NAMPT, anti- ABCA1, anti-ABCG1(abcam, 1:1000 dilution), anti-PPARα, anti-LXRα (Santa,1:500 dilution)and β-actin (Research Diagnostics) for 24 h at 4 °C. For detection of NAMPT protein in blood, the blot was stained with Ponceau S, and the region covering the 66-kDa protein was cut from the rest as a control^45^. Following 3 washes in TBST, blots were incubated with HRP-conjugated secondary antibody for 1 h. After 2 washes in TBST, the blots were scanned using the Enhanced chemiluminescence (ECL) detection system (Viagene, USA) and quantification of antigen- antibody complexes was performed using Quantity One analysis software (Bio-Rad).

### Analytical procedures

Radioactivity [3-^3^H] glucose, [1-^3^H]-cholesterol and [1-^3^H]-sterols were measured by LSC. Plasma FFA was determined spectrophotometrically using an acyl-CoA oxidase-based colorimetric kit (Wako Pure Chemical Industries, Osaka, Japan). Insulin was measured using a commercial insulin enzyme-linked immunosorbent assay kit. Triglyceride (TG), total cholesterol (TC), low-density lipoprotein cholesterol (LDL-C) and high-density lipoprotein cholesterol (HDL-C) concentrations were measured using enzymatic colorimetric methods. Plasma concentrations of apolipoprotein A-I (apoA-I) was determined with an ELISA kit. For fast protein liquid chromatography (FPLC) analysis, 100 μl pooled plasma was separated on a Superose 6 column (Amersham) at a flow rate of 0.5 ml/min as described previously^46^. Hepatic lipid contents were measured by a spectrophotometric procedure as described^47^.

### Statistical Analysis

Data were presented as means ± SE. Comparisons among groups were made using ANOVA, followed by a post hoc (PLSD) test to compare two individual groups. Differences were considered statistically significant at *P* < 0.05.

## Additional Information

**How to cite this article**: Li, S. *et al*. NAMPT knockdown attenuates atherosclerosis and promotes reverse cholesterol transport in ApoE KO mice with high-fat-induced insulin resistance. *Sci. Rep.* **6**, 26746; doi: 10.1038/srep26746 (2016).

## Supplementary Material

Supplementary Information

## Figures and Tables

**Table 1:** Metabolic parameter for HFD-fed ApoE KO mice.

**Treatment**	**PBS**	**GFP-A**	**NAMPT-A**
Body weight (g)	33.10 ± 0.95	34.56 ± 0.84	33.65 ± 1.07
FBG (mmol/L)	7.46 ± 0.33	7.25 ± 0.28	7.43 ± 0.27
FIns (mU/L)	82.99 ± 2.33	83.15 ± 2.81	85.71 ± 2.98
TC (mmol/L)	15.03 ± 0.74	15.35 ± 0.65	16.01 ± 0.61
TG (mmol/L)	1.88 ± 0.04	1.92 ± 0.07	1.96 ± 0.05
FFA (mmol/L)	2.44 ± 0.06	2.54 ± 0.07	2.49 ± 0.04
HDL-C (mmol/L)	3.34 ± 0.08	3.38 ± 0.12	4.09 ± 0.09**##
LDL-C (mmol/L)	7.63 ± 0.30	7.90 ± 0.35	7.60 ± 0.22
Hepatic TG (mg/gr tissue)	35.09 ± 1.77	34.59 ± 2.28	33.87 ± 1.99
Hepatic TC (mg/gr tissue)	5.57 ± 0.39	5.38 ± 0.38	4.30 ± 0.31*#

TC, Total cholesterol; TG, Triglyeride; FFA, Free fatty acids; HDL-C, high density lipoprotein cholesterol; LDL-C, low density lipoprotein cholesterol. PBS, HFD-fed ApoE KO mice treated with PBS; GFP-A, HFD-fed ApoE KO mice treated with Ad-GFP; NAMPT-A, HFD-fed ApoE KO mice treated with Ad-*sh*NAMPT. Data are means ± SE (n = 10 per group). *P < 0.05, **P < 0.01 *vs. P*BS group; ^#^*P* < 0.05, ^##^*P* < 0.01 *vs*. GF*P*-A group.
