# Supplementary material for: NAMPT knockdown attenuates atherosclerosis and promotes reverse cholesterol transport in ApoE KO mice with high-fat-induced insulin resistance
Source: Sci Rep. 2016 May 27;6:26746. doi: 10.1038/srep26746 (PMC4882618; doi:10.1038/srep26746)
Supplement: Supplementary Information [file srep26746-s1.pdf]

NAMPT knockdown attenuates atherosclerosis and promotes reverse cholesterol transport in ApoE KO mice with high-fat-induced insulin resistance

Shengbing Li [1,*], Cong Wang[1,*], Ke Li[1,*], Ling Li[2], Mingyuan Tian[1], Jing Xie[1], Mengliu Yang[1], Yanjun Jia[1], Junying He[1], Lin Gao[3], Guenther Boden[4], Hua Liu[5], Gangyi Yang[1]

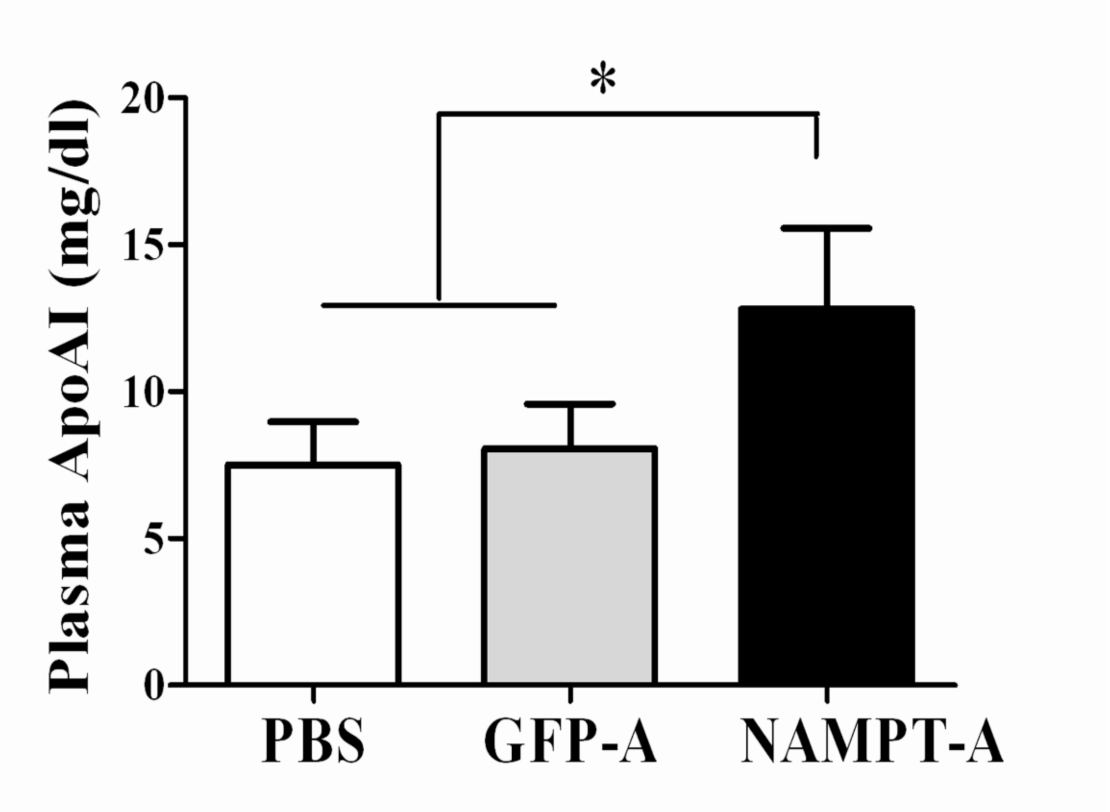

Supplemental Figure S1 NAMPT knockdown increases plasma apoA-I concentration *in vivo*. PBS, HFD-fed ApoE KO mice treated with PBS; GFP-A, HFD-fed ApoE KO mice treated with Ad-GFP; NAMPT-A, HFD-fed ApoE KO mice treated with Ad-*sh* NAMPT. HFD, high fat diet. Data are means ± S.E. * P<0.01 *vs.* PBS group.

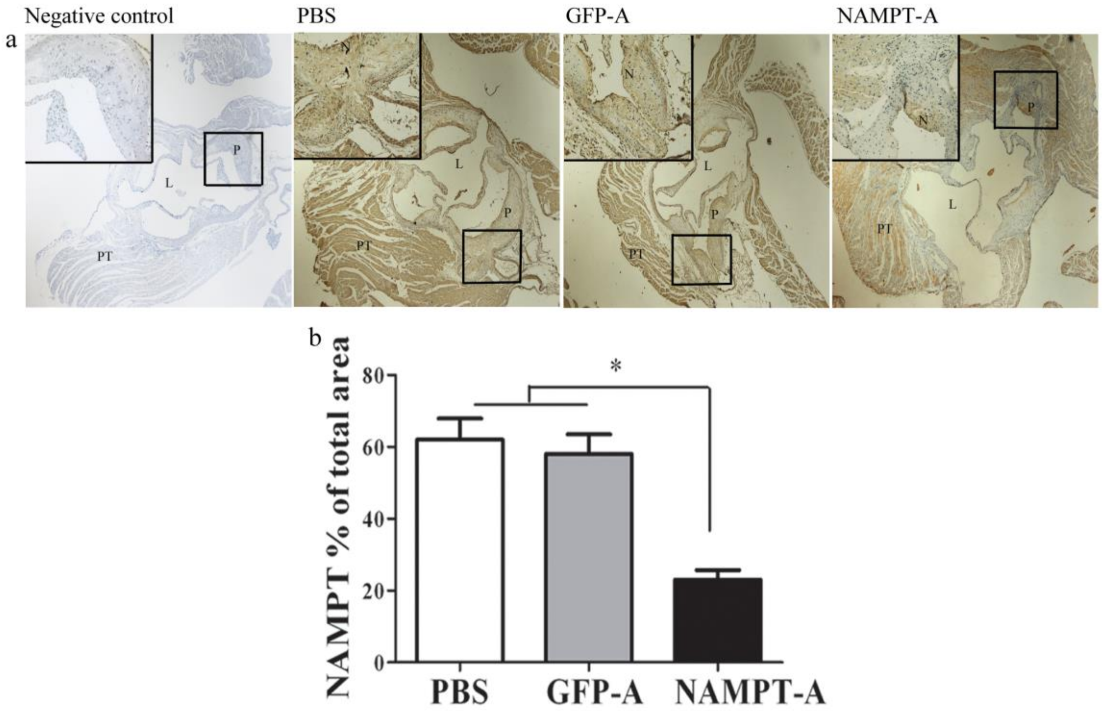

Supplemental Figure S2 Treatment with Ad-*sh*NAMPT decreases NAMPT expression in atherosclerotic lesions. (a) Representative NAMPT immunohistochemistry stained and negative control aortic root cross sections. (b) Quantitative assessment of the plaque area for NAMPT expression. PBS, HFD-fed ApoE KO mice treated with PBS; GFP-A, HFD-fed ApoE KO mice treated with Ad-GFP; NAMPT-A, HFD-fed ApoE KO mice treated with Ad-*sh* NAMPT. HFD, high fat diet; L: lumen; P: plaque; PT: perivascular tissue; N: $NAMPT^{+}$ areas. Data are the mean ±S.E. Magnification ×40 (right) and ×200 (upper left), **P* <0.01.

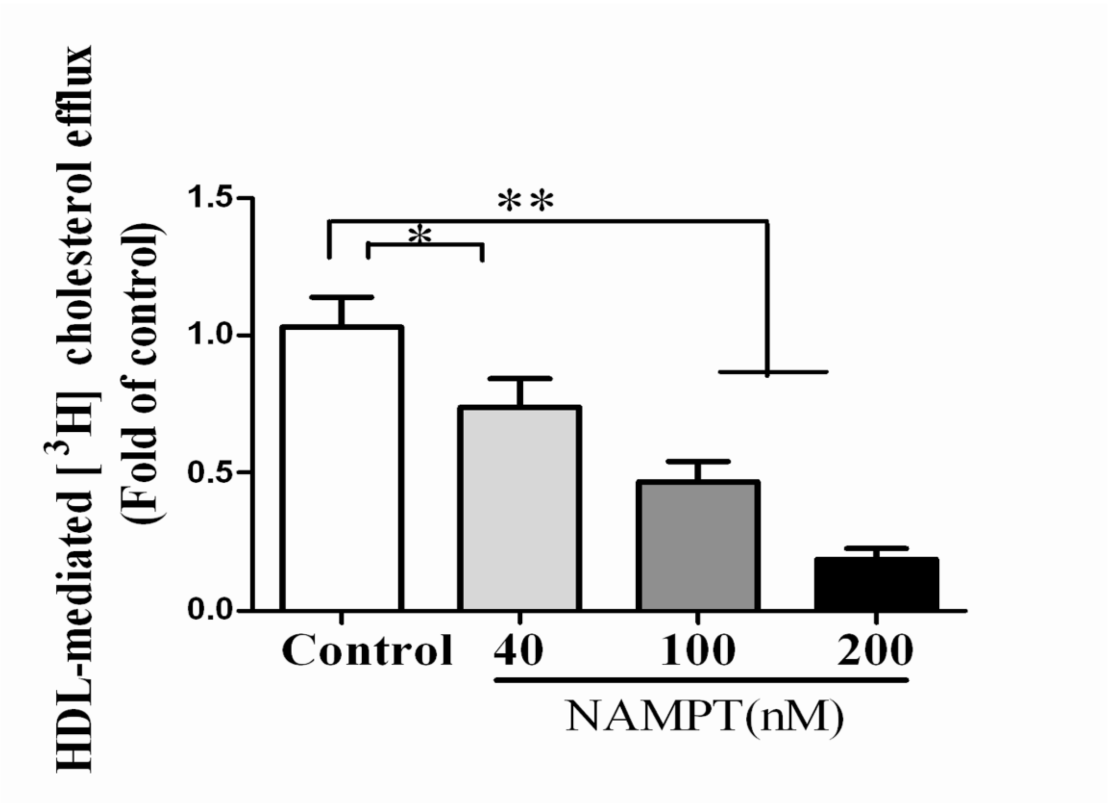

Supplemental Figure S3 NAMPTs decrease the HDL-mediated cholesterol efflux in RAW cells. ^{3}H cholesterol-loaded RAW cells were treated with NAMPT (40–200 nM) and subsequently incubated with DMEM-BSA medium with HDL (100μg /ml) as indicated. HDL-induced ^{3}H cholesterol efflux was measured as described. Values are expressed relative to the control, set as 1. Results are the mean ± S.E. of triplicate determinations, representative of three independent experiments. $^{*}P<0.05$, $^{**}P<0.01$ compared with control.

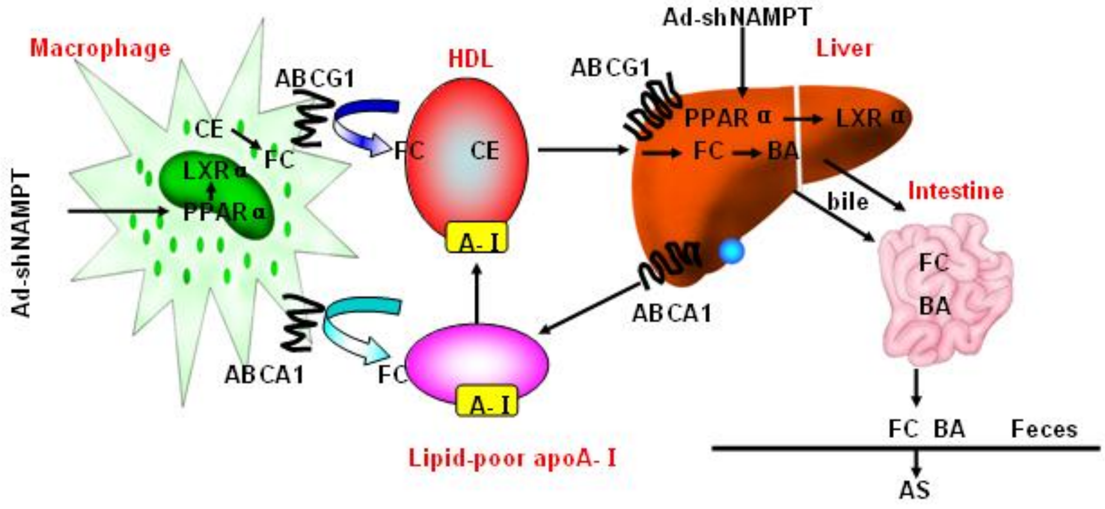

Supplemental Figure S4 Schematic representation of how Ad-shNAMPT regulates cholesterol fluxes. The administration of Ad-shNAMPT in vivo promotes the activation of PPARα-LXR pathway, in macrophages and in the liver, subsequently stimulates the expression of ABCA1/ABCG1 and in turn increases HDL-C levels and decreases hepatic TC content. On the other hand, Ad- shNAMPT also increases cholesterol excretion into feces. Finally, Ad-shNAMPT ameliorates the development of atherosclerosis.

Supplemental Table S1 Metabolic parameters for chow- or HFD-fed C57BL/6J mice

Group	NF	HF	GFP	*sh*NAMPT
Body weight(g)	27.37±0.26	35.34±0.45*	35.25±0.26*	35.31±0.29*
FBG (mmol/L)	6.28±0.14	8.21±0.14*	8.30±0.11*	8.37±0.13*
FIns (mU/L)	10.42±0.81	43.75±3.30*	42.95±2.91*	52.54±3.75*†‡
TC (mmol/L)	2.5±0.09	3.78±0.06*	3.84±0.06*	4.00±0.07*
TG (mmol/L)	0.55±0.04	0.75±0.04*	0.71±0.03*	0.73±0.04*
FFA (mmol/L)	0.48±0.02	0.89±0.04*	0.91±0.03*	0.93±0.04*
HDL-C (mmol/L)	1.72±0.03	2.10±0.05*	2.16±0.05*	2.50±0.05*††‡‡
LDL-C (mmol/L)	0.62±0.03	1.03±0.05*	1.00±0.05*	0.91±0.04*

FBG, fasting blood glucose; FIns, fasting plasma insulin; TC, Total cholesterol; TG, Triglyeride; FFA, Free fatty acids; HDL-C, high density lipoprotein cholesterol; LDL-C, low density lipoprotein cholesterol; NF, SCD-fed mice; HF, HFD-fed mice; GFP, HFD-fed mice treated with Ad-GFP; *sh*NAMPT, HFD-fed mice treated with Ad-*sh* NAMPT. Data are means ±SE. n ≥ 8 per group. $^{*}P<0.01$ *vs.* NF group; $^{\dagger}P<0.05$, $^{\dagger\dagger}P<0.01$ *vs.* HF group; $^{\ddagger}P<0.05$, $^{\ddagger\ddagger}P<0.01$ *vs.* GFP group.

Supplemental Table S2 Plasma parameters and glucose turnover data in C57BL/6J mice during insulin clamp

Index	NF		HF		GFP		*sh*NAMPT	
	Basal	Clamp	Basal	Clamp	Basal	Clamp	Basal	Clamp
FIns(mU/L)	10.42±0.81	34.89±2.13*	43.75±3.30	146.25±5.75*†	42.95±2.91	144.43±4.52*†	52.54±3.75	149.88±4.14*†
TC(mmol/L)	2.50±0.09	1.68±0.06*	3.78±0.06	2.48±0.16*†	3.84±0.06	2.51±0.15*†	3.92±0.07	2.58±0.16*†
TG(mmol/L)	0.55±0.04	0.32±0.02*	0.75±0.04	0.43±0.01*†	0.71±0.03	0.42±0.01*†	0.73±0.04	0.45±0.02 *†
FFA(mmol/L)	0.48±0.02	0.26±0.01*	0.89±0.04	0.45±0.03 *†	0.91±0.03	0.48±0.03*†	0.93±0.04	0.47±0.03 *†
HDL-C(mmol/L)	1.72±0.03	1.07±0.04 *	2.10±0.05	1.35±0.04 *†	2.16±0.05	1.30±0.04 *†	2.50±0.05	1.63±0.04*†‡
LDL-C(mmol/L)	0.62±0.03	0.42±0.03 *	1.03±0.05	0.59±0.03 *†	1.00±0.05	0.60±0.03*†	0.91±0.04	0.52±0.02*†
GIR(mg/kg/min)	---------	42.27±1.18	---------	20.50±0.77†	---------	21.12±0.71 †	---------	19.32±0.88†
GRd(mg/kg/min)	12.47±0.45	43.64±1.18*	17.51±0.51	29.74±0.52 *†	16.84±0.53	30.21±0.46 *†	18.20±0.46	28.65±0.83*†
HGP(mg/kg/min)	12.47±0.45	1.37±0.07*	17.51±0.51	9.24±0.52 *†	16.84±0.53	9.08±0.47 *†	18.20±0.46	9.33±0.85*†

TC, total cholesterol; TG, triglyeride; FFA, free fatty acids; GIR, glucose infusion rate; GRd, Glucose disposal rate; HGP, hepatic glucose production; NF, SCD-fed C57BL/6J mice; HF, HFD-fed C57BL/6J mice; GFP, HFD-fed C57BL/6J mice treated with Ad-GFP; *sh*NAMPT, HFD-fed mice treated with Ad-*sh*NAMPT. Data are means ± SE. n ≥ 8 per group. [*]P<0.01 *vs*. basal values; [†]P<0.01 *vs*. NF group; [‡]P<0.01 *vs*. HF grou

Supplemental Table S3 Characteristics of the specific primers used for RT-PCR analysis

Gene	Forward and reverse primers	Amplified	Annealing
β-actin	**5'-GCTGTCCCTGTATGCCTCT-3'**<br>**5'-GATGTCACGCACGATTTCC-3'**	220	55
NAMPT	**5'-ATTCCCGCCACAGTATCT-3'**<br>**5'-TCCCGATTGAAGTAAAGG-3'**	337	55
PPARα	**5'-TGCCTTAGAACTGGATGAC- 3'**<br>**5'-ATCTGGATGGTTGCTCTG-3'**	157	57.5
LXRα	**5'-CCTTGCTGAAGACCTCTG-3'**<br>**5'-TGGCTCTGGAGAACTCAA-3'**	174	57.5
ABCA1	**5'-AGGACGGCTTCAATCTCA-3'**<br>**5'-GGTGGCTCTTCTCATCAAT-3'**	180	57.5
ABCG1	**5'-CCAATCTCGTGCCGTATC-3'**<br>**5'-CTCTTATAGTCAGCGTCACA-3'**	160	48
